# Enhanced sensory perception and myoelectric control with high channel count implanted sensorimotor systems

**DOI:** 10.1088/1741-2552/ae24ae

**Published:** 2025-12-09

**Authors:** Sedona R Cady, Joris Lambrecht, Dustin J Tyler

**Affiliations:** 1 Department of Biomedical Engineering, Case Western Reserve University, Cleveland, OH 44106, United States of America; 2 Louis Stokes Cleveland Department of Veterans Affairs Medical Center, Cleveland, OH 44106, United States of America

**Keywords:** implanted neural interfaces, electrode channel count, myoelectric control, sensory perception

## Abstract

*Objective.* Implanted electrodes for nerve stimulation and myoelectric recording facilitate bidirectional sensory feedback and control for neuromuscular conditions such as limb loss. While increasing implanted electrode channel count offers potential benefits, it also presents engineering and implementation challenges. This case study examines how increasing implanted electrode channel count affects sensory perception and myoelectric controller performance, thereby supporting the value of these advancements. *Approach.* One participant with upper extremity transradial limb loss received a percutaneous implanted system with two 8-channel extraneural cuff electrodes on the median and ulnar nerves, totaling 16 stimulating channels. The individual later received a wirelessly connected implanted system featuring four 16-channel extraneural cuff electrodes on the median, ulnar, and radial nerves, totaling 64 stimulation channels, and four tetra-intramuscular (TIM) recording electrodes in residual muscles, totaling 32 sensing channels configured into 16 bipolar pairs. To compare sensory perception between the 16- and 64-channel stimulation systems, we assessed cumulative percept location coverage and the number of unique percept locations, estimated through hierarchical clustering. We compared performance across three myoelectric control algorithms that mapped 8, 10, or 14 intramuscular signal inputs through an artificial neural network to control a virtual hand in 4 degrees-of-freedom (DOFs), with simultaneous, independent, and proportional control. *Main results.* Increasing stimulation channel count expanded cumulative percept location coverage and increased the number of unique percept locations on the hand. Adding intramuscular recording channel inputs improved 4-DOF myoelectric control of a virtual hand, increasing target posture match percentage and path efficiency. *Significance.* This case study demonstrates that increasing the number of implanted electrodes can advance sensory restoration and myoelectric control for bidirectional upper limb prostheses. Continued development of more complex systems with higher channel counts may further improve outcomes for individuals with limb loss and enhance the function of sensorimotor restoration systems.

**Trial registration:** ClinicalTrials.gov ID: NCT04430218, 2020-06-30

## 1. Introduction

Implanted peripheral nerve interfaces can restore somatosensory feedback in upper limb neuroprostheses, enhancing the overall user experience [1]. Peripheral nerve stimulation using implanted extraneural or intraneural electrodes can elicit somatosensory percepts of varying intensities, qualities, durations, and locations in the missing hand [2–7], representing variation along the four dimensions of perception. Extraneural cuff electrodes have demonstrated stable interfaces for more than a decade [4, 8] and support long-term home use of upper limb bidirectional prostheses [1, 9].

In designing novel nerve stimulation electrodes, a key goal is to increase the number of stimulation channels to target more distinct axonal populations from varied regions of the nerve cross section, thereby generating a wider range of percept locations for sensory feedback. Perception of location depends on the set of activated axons within the nerve and the corresponding receptive fields [10]. Since axons have a somatotopic organization within the nerve [11], stimulation through each cuff electrode contact may activate a group of axons with similar receptive fields. Higher-density cuff electrodes could activate more distinct axon groups and elicit unique percept locations, which become increasingly important as the density of prosthesis force sensors increases. Supporting this hypothesis, high channel count functional electrical stimulation systems have facilitated precise muscle activation and intuitive movement restoration for individuals with paralysis [12]. For individuals with limb loss, extraneural [13, 14] and intraneural [6, 15] implanted sensory restoration systems with high channel counts enable perception of diverse sensations, but the extent to which increasing stimulation channel count impacts sensory perception remains a significant area for further research. Understanding the value of increasing stimulation channel count can guide design tradeoffs in future implanted neural interface systems.

Beyond nerve stimulation, implanted muscle electrodes are effective interfaces for sensing movement-related activity for upper limb neuroprostheses. Myoelectric signals recorded from implanted epimysial and intramuscular electrodes provide stable, high-fidelity electromyogram (EMG) inputs for advanced upper limb prosthetic controllers used by individuals with limb loss [16–21]. Earlier clinical trials demonstrated that implanted EMG-based controllers support both functional improvements and the chronic stability needed for long-term home use [18].

To improve prosthetic control, particularly for multi-joint limb movements, recording from a greater number of physiological sources may enhance decoding accuracy and enable control over more degrees-of-freedom (DOF), making prosthetic movements more closely resemble those of the sound hand. Recent advances in pattern recognition algorithms using multiple surface EMG inputs have shown improved function compared to traditional two site direct control [22]. Increasing surface EMG channel count from 8 to 16 channels enhanced controller performance in both virtual environments and with functional prostheses [23]. In parallel, innovations in high-channel-count recording systems—such as electroneurographic [24] and cortical interfaces [25]—have achieved promising results in decoding movement intent for individuals with neuromuscular conditions such as spinal cord injury and stroke. However, it remains unclear whether increasing implanted EMG electrode channel count correlates with similar improvements in myoelectric performance. Investigating this relationship is key to developing high DOF controllers that can better mimic natural control of the hand, which has approximately 20 DOFs [26].

As implanted sensorimotor interfaces become more sophisticated, increasing the number of implanted channels poses both technical and clinical challenges. Whether the added effort required to expand channel count results in meaningful performance improvements remains unevaluated. However, high channel count systems hold strong potential to significantly enhance both prosthetic control and sensory feedback perception.

In this study, we evaluated how increasing the number of implanted stimulating and sensing channels in an upper limb sensorimotor interface affects sensory perception and myoelectric control. First, we determined how increasing extraneural cuff electrode channel count influences sensory coverage and the number of unique percept locations on the hand. We hypothesized that higher channel count would increase total sensory coverage and the number of unique sensory percepts. Second, we assessed how increasing the number of intramuscular recording channels affects high DOF myoelectric controller performance. We hypothesized that increasing intramuscular recording channel count would improve controller performance.

## 2. Method

### 2.1. Study design

We compared sensory percepts reported by a study participant who received two implanted peripheral nerve stimulation systems with different extraneural cuff electrode channel counts, implanted 8 yr apart. The second system also included implanted EMG electrodes, and we examined how myoelectric controller performance metrics changed with varying EMG input counts. The participant is a male with right-sided, transradial limb loss due to a work-related traumatic injury in 2004. The participant enrolled in two study protocols, corresponding to two implanted systems. At the time of initial enrollment, he was 46 yr old and had been an experienced surface EMG myoelectric prosthesis user for 7 yr [4]. The participant was 55 yr old at the time of enrolling in the second protocol and receiving the second implanted system [13]. Both study protocols were approved under a Food and Drug Administration Investigational Device Exemption and by the Department of the Navy Human Research Protection Program. The first protocol was approved by the Veterans Affairs (VA) Northeast Ohio Healthcare System Institutional Review Board (IRB) Study #10035-H22, and the second protocol was approved by the VA Central IRB Study #19-34. The participant provided written informed consent. All research procedures were conducted in accordance with the principles of the declaration of Helsinki.

### 2.2. Implanted systems

#### 2.2.1. Percutaneous implanted system (System 1)

**Figure 1.**
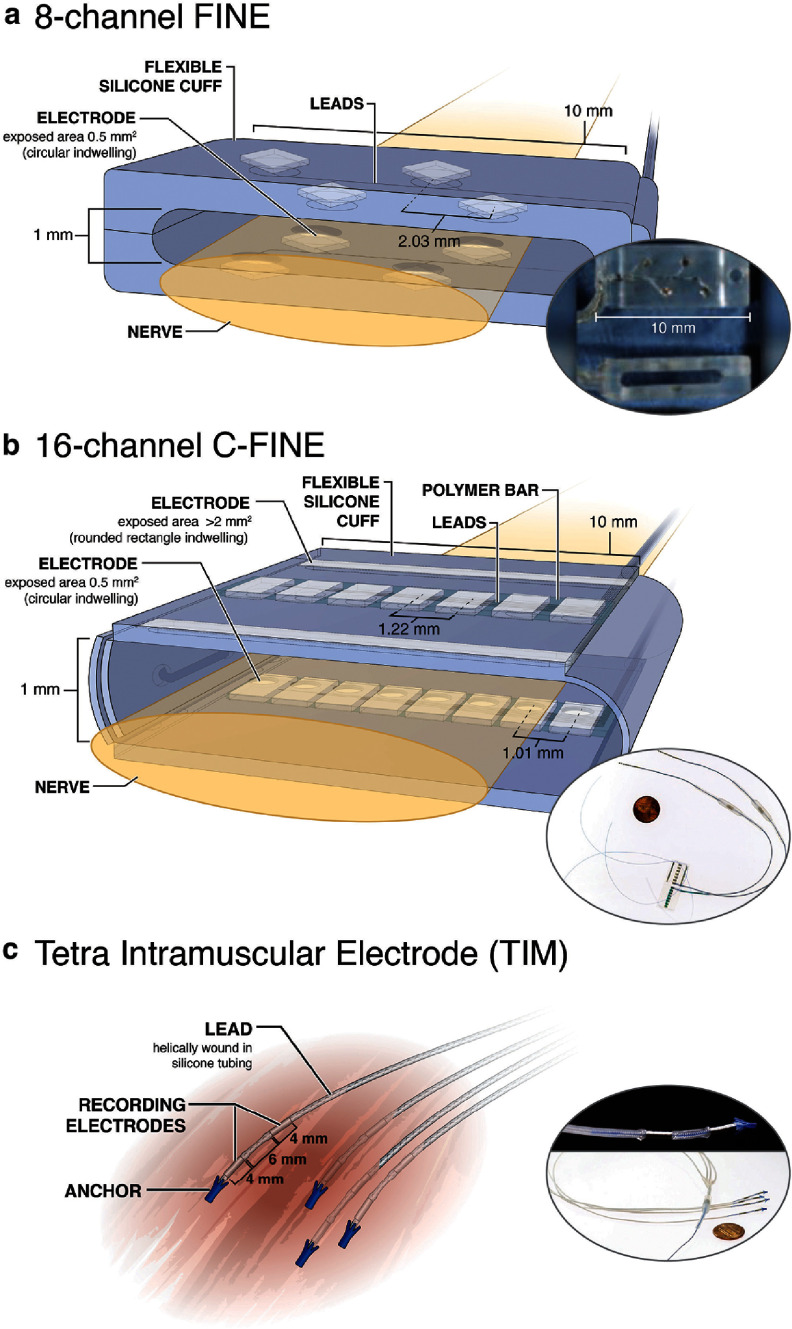
(a) 8-channel FINE, (b) 16-channel C-FINE, and (c) TIM electrode. Illustrations (left) and photographs (right) show electrode designs. All nerve cuffs for both systems were implanted in the mid-upper arm proximal to the elbow. Radial nerve cuffs were placed in the same locations for both systems, and the distal median nerve 16-channel C-FINE in System 2 was at the same location as the median nerve 8-channel FINE in System 1. A second 16-channel C-FINE was placed proximally on the median nerve in System 2. FINE and C-FINE contacts had 0.5 mm^2^ exposed circular areas of platinum-iridium. FINEs had intraluminal areas of 10 × 1.0 mm and 10 × 1.5 mm for the median and radial nerves, respectively, and C-FINEs had intraluminal areas of 10 × 1.5 mm for median nerve cuffs and 10 × 1.0 mm for ulnar and radial nerve cuffs. System 2 TIM electrodes were implanted in muscles distal to the elbow in the residual limb for bipolar EMG recording, and each bipolar pair consisted of two 316LVM stainless steel contacts spanning 4 mm and separated by 6 mm [29]. The photo shown in (a) is from [4], reprinted with permission from AAAS.

The percutaneous peripheral nerve stimulation system, previously described [4], included two accessible 8-channel flat interface nerve electrodes (FINEs) [27] implanted on the median and radial nerves in the mid-upper arm proximal to the elbow, totaling 16 stimulation contacts (figure 1(a)). Intraluminal cross-sectional areas were 10 × 1.0 mm and 10 × 1.5 mm for the median and radial nerve FINEs, respectively. The FINEs connected to percutaneous leads via spring-and-pin connectors [28], and the percutaneous leads exited the mid-upper arm. This system did not include implanted EMG electrodes. The participant maintained the percutaneous system for 8 yr during his research participation [4].

#### 2.2.2. Wirelessly connected implanted system (System 2)

The participant received the implanted Somatosensory Electrical Neurostimulation and Sensing (iSens®) system in a surgical procedure described previously [13, 14]. Designed nearly a decade after the percutaneous system, the iSens system included several design improvements to enable wireless communication and simultaneous nerve stimulation and myoelectric recording. The system included four 16-channel composite FINEs (C-FINEs) [30] implanted in the mid-upper arm, including two on the median nerve (10 × 1.5 mm), one on the ulnar nerve (10 × 1.0 mm), and one on the radial nerve (10 × 1.0 mm) to total 64 stimulation channels. Each C-FINE had 15 evenly spaced 0.5 mm^2^ contacts and one >2 mm^2^ contact, consisting of two electrically connected areas, which was used as the anode for bipolar stimulation (figure 1(b)). Four TIM electrodes [29] were implanted distal to the elbow in residual limb muscles (figure 1(c)), including extensor digitorum communis (EDC), supinator, extensor carpi radialis longus (ECRL), extensor carpi radialis brevis (ECRB), extensor carpi ulnaris (ECU), flexor pollicis longus (FPL), pronator teres, flexor carpi radialis (FCR), flexor carpi ulnaris (FCU), and flexor digitorum superficialis (FDS), providing 16 bipolar EMG channels. EDC and FDS contained four TIM bipolar channels each, whereas all other muscles contained one channel each. C-FINEs and TIMs connected to two Smart Stim and two Smart Sense Leads, respectively, which controlled internal processing of stimulation and EMG, and the Smart Leads connected to an implanted neural controller (INC) via bifurcated leads [13]. The INC powered the implanted components and communicated via Bluetooth Low Energy (BLE) to an external Hub. A PC that connected to the Hub controlled sensory and motor experiments.

### 2.3. Experiment 1: sensory perception

#### 2.3.1. Sensory data reporting periods

The two sensory restoration systems were labeled as 16-channel (percutaneous) and 64-channel (iSens). Sensory percepts were reported during three sessions per system: at 6-, 21-, and 34 months post-implant for the 16-channel system, and at 2 weeks, 1 month, and within 6–10 months post-implant for the 64-channel system. The 64-channel system was implanted 8 yr after the original 16-channel system, separating data collection by approximately 8 yr.

#### 2.3.2. Peripheral nerve stimulation and percept reporting

The percutaneous and iSens systems delivered electrical stimuli via extraneural cuff electrodes, with each stimulation contact tested separately. Electrical stimuli were charge-balanced, biphasic, cathode-first pulse trains. For the percutaneous system, we tested monopolar stimuli with a rectangular cathodic phase and exponentially decreasing anodic phase. For the iSens system, we implemented bipolar stimuli with rectangular cathodic and anodic phases. Sensory percepts were recorded at sensory detection threshold, defined as the minimum charge required for perception. Stimulation was delivered at 100 Hz with pulse widths of 255 *μ*s for the percutaneous system and 250 *μ*s for the iSens system. Two percutaneous sessions used a 1 Hz sinusoidal modulation envelope of pulse width [4], while all other sessions used constant pulse widths. Percepts at sensory detection threshold were determined by increasing pulse amplitude using a staircase method in steps of 0.1 mA (percutaneous) or 0.01 mA (iSens) until the participant detected a sensation. Step sizes were chosen due to differences in parameter resolutions. Next, we decreased pulse width via a binary search to find the lowest value within 5 *μ*s (percutaneous) or 10 *μ*s (iSens) at which the participant could report sensation [8]. If the participant did not feel the stimulus, the pulse width was increased halfway up the remaining range; if they did feel it, the pulse width was decreased halfway. This continued until finding the detection threshold within 5 *μ*s or 10 *μ*s accuracy, based on each system’s parameter resolutions. Upon establishing sensory detection threshold perception, the participant drew perceived location on a template image of the hand using a pen and paper or a stylus and touchscreen monitor (Cintiq Pro 24, Wacom International, USA). The participant freely reported as many quality descriptors as needed to define the sensation quality. A single trial was performed per stimulation contact during each session to avoid sensory adaptation and reduce experimental time.

#### 2.3.3. Percutaneous implanted system stimulation setup

A custom Universal External Control Unit (UECU) (Cleveland Functional Electrical Stimulation Center, Cleveland VAMC, Cleveland, Ohio, USA), connected to a power isolation station, delivered current-controlled stimulation to FINEs in the percutaneous system [4]. Stimulation parameters were selected via a custom MATLAB-Simulink model (The MathWorks Inc., Natick, MA, USA), and parameters used to elicit percepts at sensory detection threshold were in the following ranges: pulse widths between 0–255 *μ*s (1 *μ*s resolution), pulse amplitudes between 0–1 mA (0.1 mA resolution), and 5 s pulse trains. A surface electrode on the dorsal arm near the elbow served as the return.

#### 2.3.4. Wirelessly connected implanted system stimulation setup

Custom MATLAB interfaces automated communication to the Hub and controlled set stimulation parameters. Single-contact stimulation consisted of setting each 0.5 mm^2^ electrode contact as the cathode, totaling 60 contacts across the four C-FINEs. The >2 mm^2^ contact strips for each corresponding C-FINE served as the anodes for bipolar stimulation, and the anodic phase was half the amplitude and twice the width of the cathodic phase. Stimulation parameters used to elicit percepts at sensory detection threshold included pulse widths between 0–250 *μ*s (10 *μ*s resolution), pulse amplitudes between 0–1 mA (0.01 mA resolution), and 3 s pulse trains.

#### 2.3.5. Perceived location coverage analysis

Each perceived location drawing was represented as a two-dimensional binary image, and only locations distal to the wrist were included in the analysis. For each system, we computed the cumulative coverage of perceived locations distal to the wrist as the union of all location drawings. Mean single-contact perceived area was calculated as the percentage of total hand area and scaled to the size of the participant’s sound hand (wrist-middle-finger length: 19.0 cm). Percepts were categorized as tactile, proprioceptive, and/or painful based on the participant’s reported sensation quality descriptors. We computed the cumulative coverage of perceived locations for tactile-only percepts to exclude any large, whole-hand sensations associated with proprioceptive percepts. We also computed cumulative location coverage for proprioception-only and pain-only percepts.

#### 2.3.6. Unique percept location model

The model included tactile and/or proprioceptive percepts and excluded painful percepts to prioritize sensations that could be associated with prosthesis sensors. Percept uniqueness was determined by comparing location dissimilarity *D*, calculated as the complement of the Jaccard similarity between two binary location images *L_1_* and *L_2_* (equation (1)). Jaccard similarity measures image overlap, analogous to overlapping perceptive fields [14], and it is defined as the intersection divided by the union of locations,

\begin{align*}D = 1 - \frac{{\left| {{L_1}{{\mathop \cap \nolimits}}{L_2}} \right|}}{{\left| {L1{{\mathop \cup \nolimits}}{L_2}} \right|}}.\end{align*}

For each system and session, we applied complete-linkage hierarchical clustering to locations reported from single-contact stimulation. Dissimilarity between each pair of percept locations determined the multilevel similarity hierarchy, represented as a linkage tree dendrogram, and setting a dissimilarity ‘cutoff’ split the hierarchy into unique percept clusters. Location dissimilarities of 1 and 0 represented nonoverlapping and identical percepts, respectively. We used dissimilarity cutoffs between 0.01 and 1 in increments of 0.01. The resultant cluster counts estimated the number of unique percept locations for each system. While the location dissimilarity cutoff could be set at any value between 0–1, we highlighted examples of unique percept counts between the 16-channel and 64-channel systems at dissimilarity cutoffs of 0.7 and 0.9 based on prior percept stability results showing stable and shifting percept locations across infrequent experimental sessions [14]. Percept locations that scored as more dissimilar than the 0.7–0.9 range would be more likely to be classified as unique percepts, whereas dissimilarities less than the 0.7–0.9 range would more likely be classified as the same percept. We repeated the clustering method to compare unique percept locations between the radial nerve’s 8-channel FINE and 16-channel C-FINE, the median nerve’s 8-channel FINE and two 16-channel C-FINEs, and between the two 16-channel C-FINEs on the median nerve.

### 2.4. Experiment 2: myoelectric controller performance

#### 2.4.1. Motor data reporting periods

EMG training data were collected during a single session, 2.6 yr after iSens implantation, and the training data were used to build three myoelectric controllers tested in experimental sessions 1, 3, and 5 months later.

#### 2.4.2. Intramuscular EMG collection

The iSens system enabled recording intramuscular EMG via TIM electrodes. Two Smart Sense Leads converted 16 channels of raw EMG to processed EMG features, including waveform length (WFL) [31], at 10-bit resolution and a 50 ms update rate. WFL, computed as the average absolute difference between successive EMG samples from 1 kHz raw EMG across 100 ms windows [31], was chosen because it was not affected by inconsistent baseline EMG activity, as previously shown [14]. The INC transmitted EMG data to the Hub and PC for control algorithms.

#### 2.4.3. Controller training data collection

A custom MATLAB-Simulink interface automated EMG WFL data collection from 16 channels and prompted the participant to perform 10 repetitions of 32 hand postures (320 trials) [16, 17, 32]. Postures included all single and paired movements across the following four DOFs: wrist pronation–supination, wrist flexion–extension, thumb abduction/extension–adduction/flexion, and index/middle/ring/pinky finger flexion–extension. A four-element vector represented each posture to indicate negative (−1), neutral (0), or positive directions (+1) per DOF. Each trial captured 5 s of EMG at 20 Hz: 2 s of relaxation, 2 s of moving to the prompted posture, and a final 1 s of relaxation. For each of the 32 training postures, we excluded one repetition that displayed the least similar EMG activity patterns compared to the other nine repetitions.

#### 2.4.4. Artificial neural network (ANN) controller development

We developed three ANN myoelectric controllers [32–35], differing in the number of EMG channel inputs. We excluded two of the 16 EMG channels, both implanted in FDS, due to noise-only recordings observed during voluntary muscle contractions. The first controller utilized the remaining 14 channels (table 1). The second controller excluded redundant channels implanted in FDS and EDC, such that only one channel was implanted in each muscle, totaling 10 channels. The third controller further excluded channels implanted in FPL and ECRB to mimic muscle inputs previously used to build 3- and 4-DOF TIM-based controllers [17].

**Table 1.** Anatomical locations of intramuscular EMG channels used for each myoelectric controller. TIM electrodes were implanted in the FPL, pronator teres, FCR, FCU, FDS, EDC, supinator, ECRL, ECRB, and ECU muscles. Of the 14 responsive EMG channels, controllers excluded 0, 4, or 6 channels to predict joint angle velocity in 4 DOFs.

Muscle	14-ch controller	10-ch controller	8-ch controller
FPL	✓	✓	Exclude
Pronator teres	✓	✓	✓
FCR	✓	✓	✓
FCU	✓	✓	✓
FDS	✓	✓	✓
FDS	Exclude	Exclude	Exclude
FDS	✓	Exclude	Exclude
FDS	Exclude	Exclude	Exclude
EDC	✓	✓	✓
EDC	✓	Exclude	Exclude
EDC	✓	Exclude	Exclude
EDC	✓	Exclude	Exclude
Supinator	✓	✓	✓
ECRL	✓	✓	✓
ECRB	✓	✓	Exclude
ECU	✓	✓	✓

User effort was estimated as the mean WFL across selected EMG channels at each moment in time [16], and the product of normalized user effort and the 4-element posture direction vector created 4-element effort vectors that comprised the outputs for controller training [16]. The first and last second of each 5-s trial were excluded. The inputs for controller training consisted of the 14-, 10-, or 8-channel EMG WFL inputs at each moment in time, and the outputs consisted of the corresponding 4-element effort vectors. Data timepoints were divided into training (70%), validation (15%), and test (15%) sets, and we developed ANNs that had one hidden layer with the same number of nodes as the input layer using MATLAB’s machine learning toolbox.

#### 2.4.5. Posture matching controller evaluation

The trained ANNs were used to map 14, 10, or 8 EMG channels to joint angle velocity in 4 DOFs for online evaluation. Prior to evaluation, the participant freely moved a virtual hand using each controller, and we adjusted joint angle velocity gains and thresholds based on preference. For each controller, the participant matched 80 pseudo-randomly generated 4-DOF target postures displayed on a virtual 3-dimensional limb on a PC monitor [16, 17, 32]. The left, contralateral-side hand presented the target posture, and the right, residual-limb-side hand freely moved in 4 DOFs based on voluntary movements. A successful trial required holding the hand within 15% of all target posture joint angles for 1 s within the 30 s trial limit. Outcome metrics included match percentage, time-to-target, and path efficiency [16, 17, 36]. The participant completed two sessions for the 14-channel controller and three sessions each for the 10- and 8-channel controllers. We determined mean time-to-target and path efficiency across sessions per target posture, excluding unsuccessful trials. The participant was blinded to controller type.

### 2.5. Statistical analysis

All analyses were completed in MATLAB. For sensory analyses, including cumulative location coverage and location cluster count, we compared results between each system qualitatively due to the nature of the single case study’s low sample size and the large time lapse—approximately 8 yr—between data sets for each nerve stimulation system. For motor control analyses, significance thresholds were set to *α* = 0.05. Fisher’s exact tests compared match percentages between controllers. One-way ANOVAs with Tukey’s post-hoc pairwise adjustments compared time-to-target and path efficiency. Outcomes for both sensory and motor control results were reported as mean ± standard error.

## 3. Results

### 3.1. Sensory perception results

#### 3.1.1. Increased channel count expanded perceived location coverage

**Figure 2.**
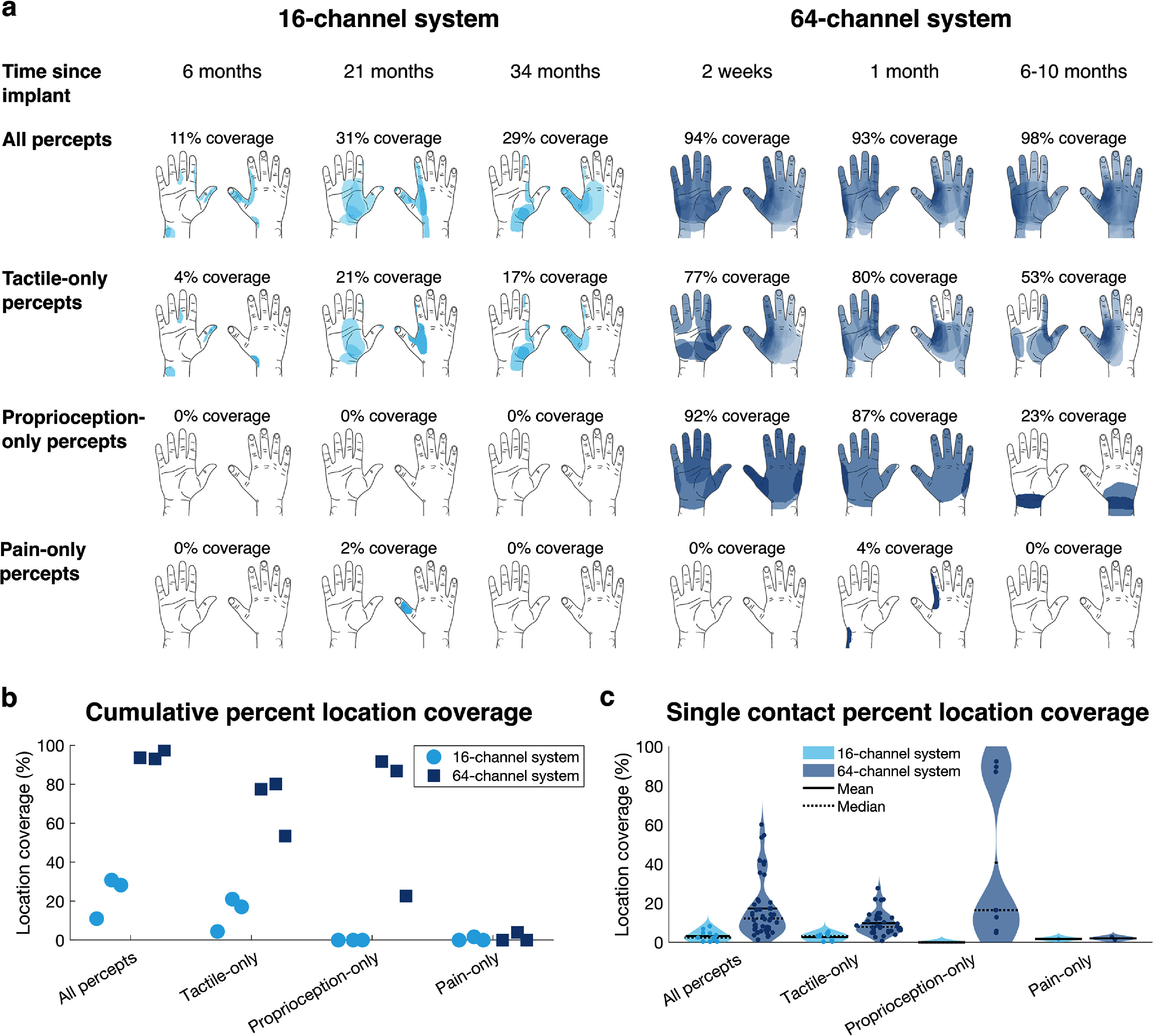
(a) Cumulative location coverage of the hand, calculated as the union of percepts evoked by single-contact stimulation, using the 16-channel (left) and a 64-channel (right) nerve stimulation systems. Coverage is shown for all percepts, tactile-only percepts, proprioception-only percepts, and pain-only percepts. Shading opacity indicates the percent of contacts evoking percepts at each pixel. (b) Session-wise cumulative location coverage for each system, representing the same data shown in (a). (c) Location coverage from individual FINE or C-FINE contacts in each system. Individual points represent location coverage per contact averaged across three sessions, and the violin plots show the distribution.

The 64-channel system produced greater cumulative hand coverage than the 16-channel system for all percepts (mean ± SE across three sessions per system: 95 ± 1% vs. 23 ± 6%) and for tactile-only percepts (70 ± 9% vs. 14 ± 5%) (figures 2(a) and (b)). Large and whole-hand percepts associated exclusively with proprioceptive quality descriptors were reported with the 64-channel system (67% ± 22%) but not the 16-channel system (0% ± 0% across all sessions) (figures 2(a) and (b)). Both systems elicited only one pain-only percept, each during a single session (figure 2(a)).

Single-contact percept areas were larger in the 64-channel system (all percepts: 17% ± 15% vs. 3% ± 3%, estimated as 65.0 ± 57.0 cm^2^ vs. 12.1 ± 10.1 cm^2^; tactile-only percepts: 10% ± 6% vs. 3% ± 2%, 37.0 ± 22.9 cm^2^ vs. 10.0 ± 7.2 cm^2^; proprioception-only percepts: 41% ± 41% vs. 0% ± 0%, 153.6 ± 154.1 cm^2^ vs. 0 ± 0 cm^2^), contributing to the increased hand coverage (figure 2(c)). Pain-only percepts from both systems were similar in area (64- vs. 16-channel systems: 2% ± 1% vs. 2% ± 0%, 7.6 ± 3.5 cm^2^ vs. 6.5 ± 0.0 cm^2^). The single pain-only percept from the 64-channel system was described as ‘pain,’ ‘uncomfortable,’ ‘burning,’ and ‘stinging’ [14], whereas the 16-channel system elicited one described as a ‘toothache pain.’

All contacts in the 16-channel system evoked percepts, averaging 11 percepts on the hand and 5 percepts, all from the radial nerve FINE, on the residual limb upper arm. In the 64-channel system, 52 of 60 tested contacts evoked percepts, averaging 40 percepts on the hand and 12 percepts—5 from the ulnar nerve C-FINE and 7 from the radial nerve C-FINE—on the residual limb. Upper arm percepts from radial nerve stimulation were notably associated with contractions. While either constant stimulation or sinusoidal pulse width modulation was applied for the percutaneous system at different sessions, no changes in location or area of sensation were reported.

#### 3.1.2. Increased channel count created more unique sensory percepts

**Figure 3.**
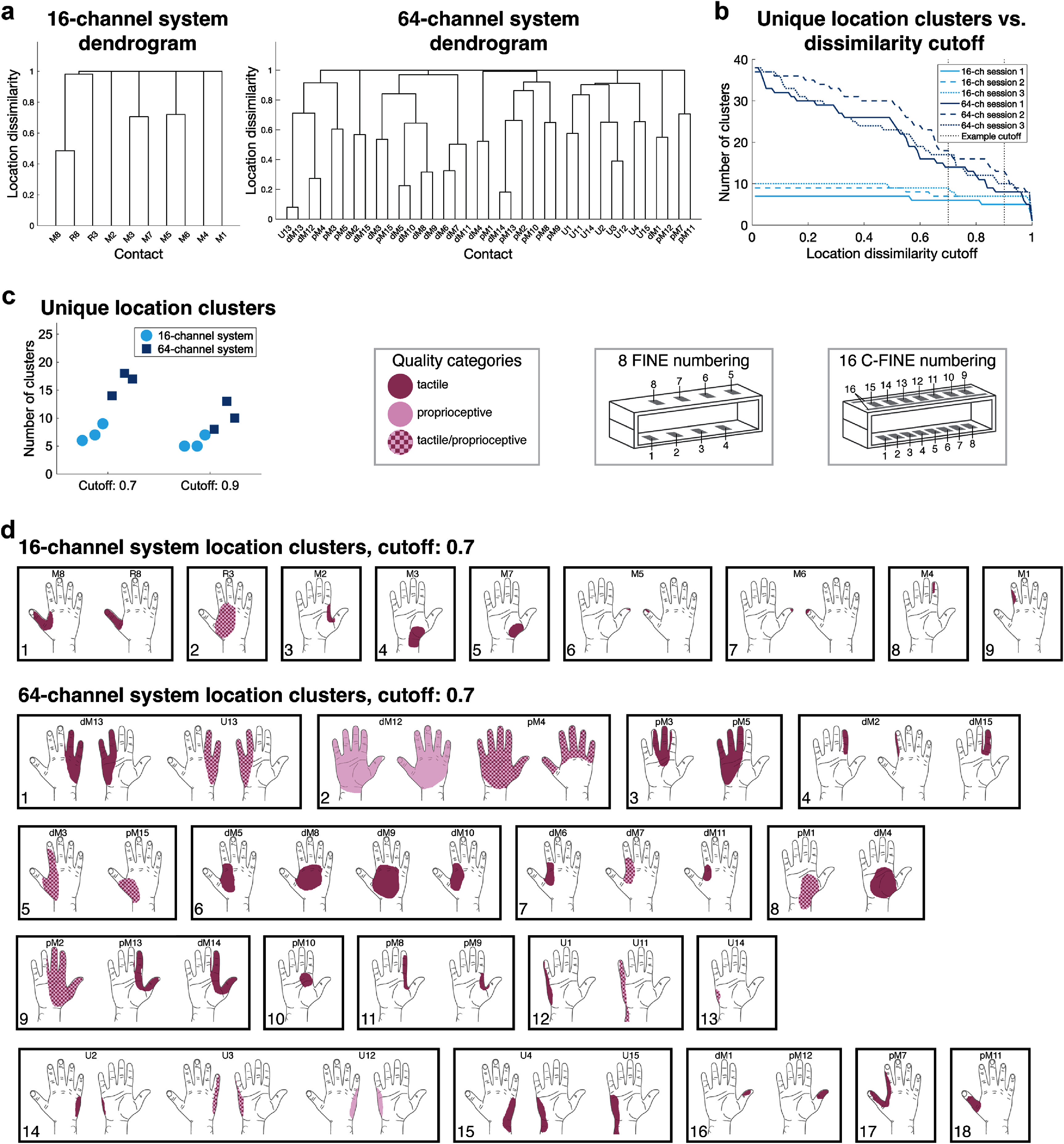
(a) Example dendrograms from the sessions with the greatest overall cluster counts per nerve stimulation system, corresponding to the highest number of unique percept locations: session 3 for the 16-channel system (left) and session 2 for the 64-channel system (right). (b) Cluster count, estimating unique percept locations, as a function of location dissimilarity cutoff for each system and session. Note that the example sessions shown in (a)—session 3 for the 16-channel system and session 2 for the 64-channel system—had slightly higher cluster counts than other sessions across location dissimilarity cutoffs, though overall cluster counts remained relatively consistent across sessions for each system. (c) Cluster counts per system and session for example dissimilarity cutoffs of 0.7 and 0.9. (d) Unique percept clusters (0.7 cutoff) for the 16-channel system during session 3 (top) and the 64-channel system during session 2 (bottom). Color shading indicates categorized qualities. Labels above each percept location indicate stimulation contacts, with percepts spanning both sides of the hand having labels above and centered between the palm side and back side drawings.

Dendrogram cluster trees represented location dissimilarities between pairs of percepts evoked from single-contact stimulation, where 0 indicated identical and 1 indicated non-overlapping locations (figure 3(a)). The 64-channel system produced more complex dendrogram branches due to variable location dissimilarities and more instances of lower dissimilarities due to overlapping percepts. Applying a location dissimilarity cutoff across each dendrogram defined whether two percept locations were unique or the same percept.

Hierarchical clustering of non-painful percepts showed higher cluster counts in the 64-channel system at all location dissimilarity cutoffs (figure 3(b)). As the location dissimilarity cutoff increased, cluster count steadily decreased for the 64-channel system, whereas the 16-channel system remained at a stable cluster count through a cutoff of approximately 0.5 before decreasing at a slower rate than the 64-channel system. Larger percept areas and overlapping locations in the 64-channel system contributed to the steeper decrease in cluster count.

At a location dissimilarity cutoff of 0.7, the 16-channel system produced up to 9 clusters (7.3 ± 0.9, mean ± SE), while the 64-channel system produced up to 18 clusters (16.3 ± 1.2, mean ± SE) (figures 3(c) and (d)). Increasing the location dissimilarity cutoff to 0.9 reduced cluster counts to 5.7 ± 0.7 and 10.3 ± 1.5 clusters, respectively (figure 3(c)). For both cutoffs, the 64-channel system demonstrated more unique location clusters.

**Figure 4.**
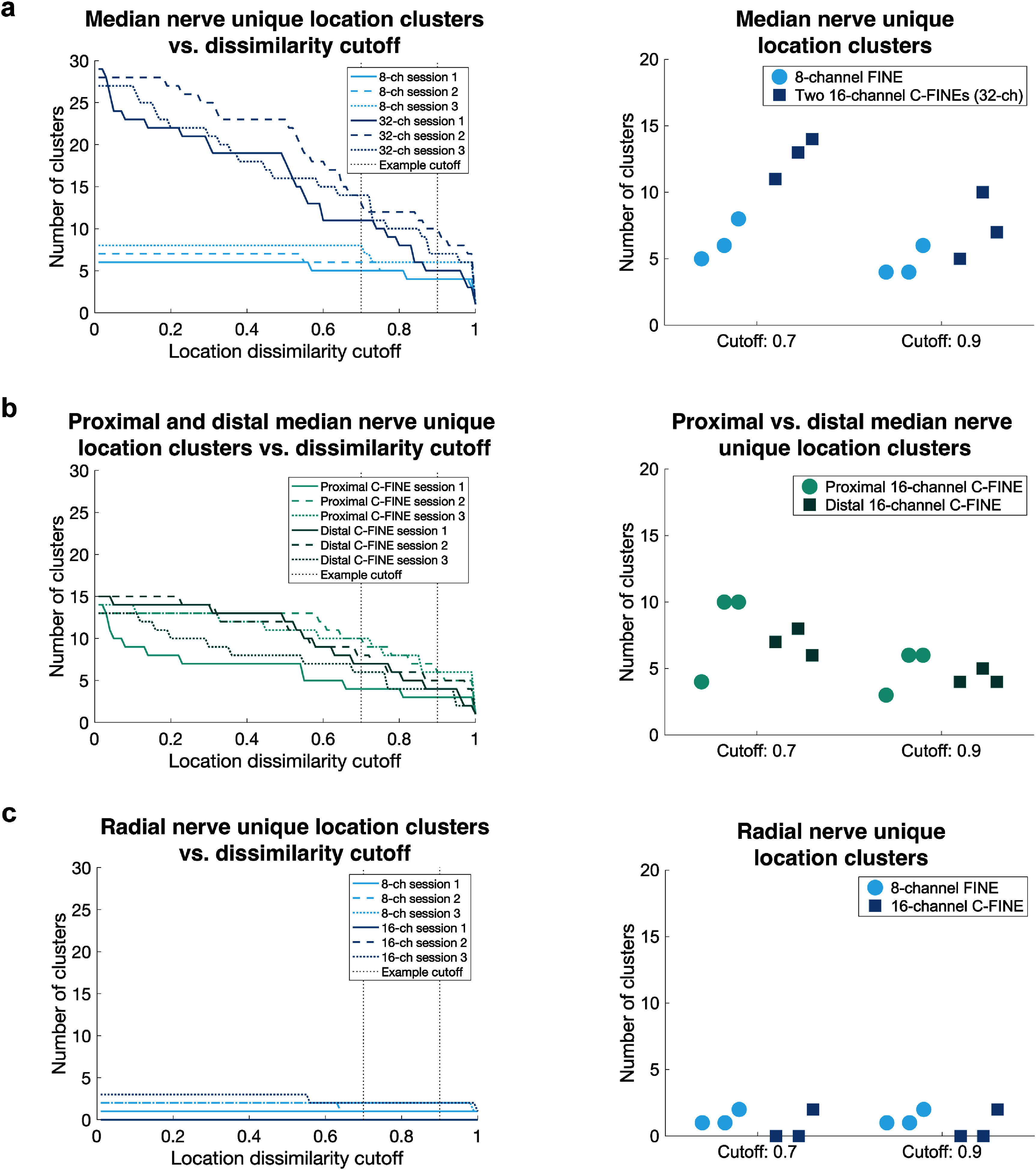
Unique percept cluster count as a function of location dissimilarity cutoff (left) and at cutoffs of 0.7 and 0.9 (right) during three sessions each for: (a) one 8-channel FINE vs. two 16-channel C-FINEs (32 channels) on the median nerve, (b) proximal vs. distal 16-channel C-FINEs on the median nerve, and (c) one 8-channel FINE vs. one 16-channel C-FINE on the radial nerve.

In addition to investigating the effects of increasing overall channel count across multiple nerves, we also examined how increasing stimulation channel count on a single nerve affected unique percept generation. Upgrading the median nerve interface from one 8-channel FINE to two 16-channel C-FINEs increased unique percept location clusters (figure 4(a) *left*). At a dissimilarity cutoff of 0.7, 32 channels produced more unique percept location clusters (12.6 ± 0.9) than 8 channels (6.3 ± 0.9) (figure 4(a) *right*). Cluster counts converged at a dissimilarity cutoff of 0.84, such that higher cutoffs resulted in approximately the same number of clusters. Applying a dissimilarity cutoff of 0.9 resulted in a small increase in cluster count for the 32-channel setup (4.6 ± 0.7 vs. 7.3 ± 1.5 clusters) (figure 4(a) *right*). The two 16-channel median nerve C-FINEs in the iSens system produced similar cluster counts across all cutoffs, including at example cutoffs of 0.7 or 0.9 (figure 4(b)).

Radial nerve stimulation through the 8-channel FINE and 16-channel C-FINE produced low cluster counts across all dissimilarity cutoffs (figure 4(c) *left*). Upgrading from an 8-channel FINE to 16-channel C-FINE did not affect the number of unique percepts in the hand for either 0.7 or 0.9 example cutoff values (figure 4(c) *right*). Most radial nerve percepts were on the residual limb rather than the hand. During two sessions, the 16-channel radial nerve C-FINE evoked only residual limb percepts; in the third session, 5 out of 15 contacts evoked hand percepts, three of which were non-painful. Contacts in the 16-channel C-FINE that did not elicit sensation below the charge safety limit were adjacent to each other on one side of the cuff, suggesting that the nerve was not centered in the C-FINE.

### 3.2. Myoelectric control results

#### 3.2.1. Increased intramuscular EMG channel count improved controller performance

**Figure 5.**
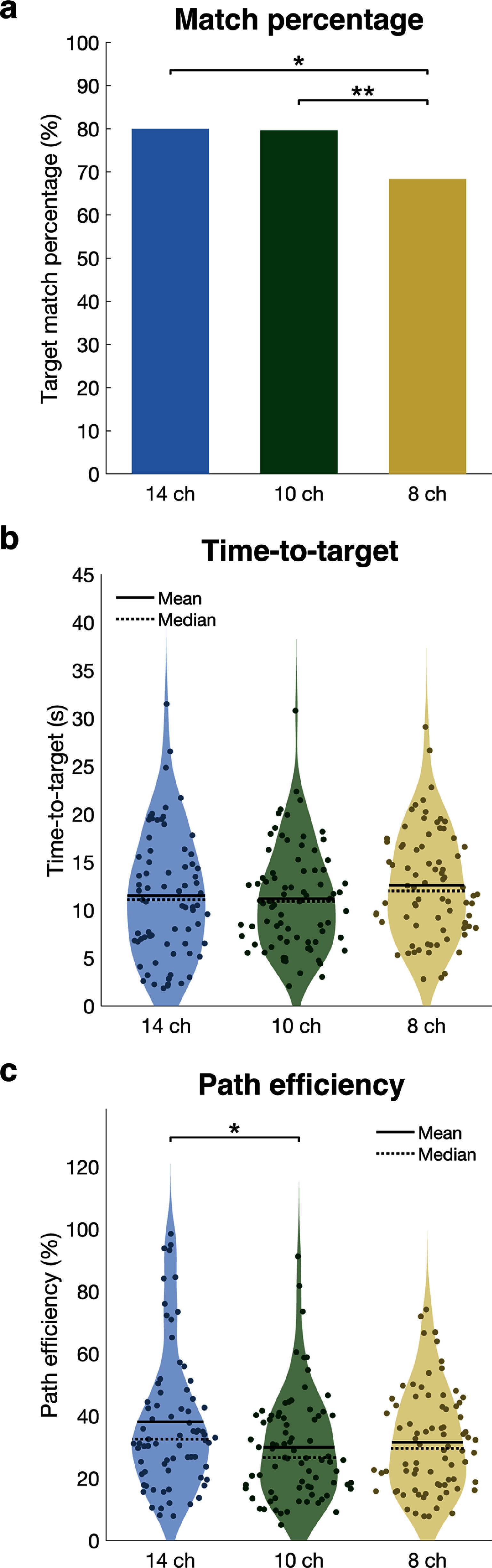
Posture matching task outcome measures using 4-DOF controllers with 14, 10, or 8 intramuscular EMG inputs to an ANN, tested in a virtual environment. (a) Match percentage, or the number of successful trials out of the total number of trials across all experimental sessions. (b) Time-to-target, or the time to reach a matched target posture. (c) Path efficiency, or the Euclidean distance between the start position and the matched posture position divided by the total distance traveled during the trial in joint angle space. The average time-to-target and path efficiency for each target posture (n = 80 postures) are shown, averaged across three sessions (*p < 0.05, **p < 0.01).

Control of a virtual limb in 4 DOFs showed higher posture matching success rates for 14- and 10-channel controllers (80% each) compared to the 8-channel controller (68%) (Fisher’s exact test, 14 vs. 8 channels: *p* = 0.0084; 10 vs. 8 channels: *p* = 0.0050) (figure 5(a)). Additional EMG inputs from FPL and ECRB muscles in the 14- and 10-channel controllers likely contributed to improved decoding accuracy, while redundant FDS and EDC inputs in the 14-channel controller did not significantly improve match percentage compared to the 10-channel controller (Fisher’s exact test, *p* = 1.0).

Time-to-target for matched trials was similar across controllers (14-channel: 11.3 ± 0.7 s; 10-channel: 11.6 ± 0.6 s; 8-channel: 12.4 ± 0.6 s) (figure 5(b)). The 8-channel controller trended on average 0.8-1.1 s slower than the other controllers, but time-to-target did not significantly differ (ANOVA with Tukey’s post-hoc pairwise adjustment, 14- vs. 10-channel: *p* = 0.94; 14- vs. 8-channel: *p* = 0.49; 10- vs. 8-channel: *p* = 0.70). Additionally, the 8-channel controller had more unmatched trials that terminated at the 30 s trial limit, suggesting even slower performance.

Matched trial mean path efficiencies were 38 ± 3% (14-channel), 30 ± 2% (10-channel), and 32 ± 2% (8-channel), with the 14-channel controller significantly outperforming the 10-channel controller and trending higher than the 8-channel controller (ANOVA with Tukey’s post-hoc pairwise adjustment, 14- vs. 10-channel: *p* = 0.021; 14- vs. 8-channel: *p* = 0.094; 10- vs. 8-channel: *p* = 0.83) (figure 5(c)). Additional FDS and EDC inputs likely contributed to the 14-channel controller’s improved path efficiency compared to the 10-channel controller, which did not have the same redundant inputs. The exclusion of unmatched trials likely biased 8-channel controller path efficiency upward due to its overall lower match percentage.

## 4. Discussion

### 4.1. Sensory perception

In this case study, increasing the number of implanted stimulating channels expanded sensory coverage across the hand, both overall and for percepts only associated with tactile qualities. The ability to create percepts across different parts of the hand is particularly important for integration with multi-sensor bidirectional prostheses.

The overall increase in location coverage resulted from two factors. First, the higher electrode contact density likely enabled activation of more axon populations with differing receptive fields. Second, while percepts elicited from both systems had larger areas than the smallest receptive fields of skin mechanoreceptors [37, 38], percepts produced by the 16-channel C-FINEs in the iSens system had larger areas than the 8-channel FINEs in the percutaneous system. The number of axons recruited, the receptive field sizes, or both factors may have contributed to the larger percept areas. The cuff electrodes in both systems had contacts of the same exposed area, shape, and material, but the difference in percept areas signifies a difference in the electric field generated within the nerve fascicles. Although previous testing demonstrated that the reshaping force of the C-FINE matched that of the FINE [30], the increased flexibility of the C-FINE could impact nerve reshaping and selectivity in practice. Sensory detection thresholds reported with the participant’s distal median and radial nerve 16-channel C-FINEs, approximately 170 nC [14], exceeded the thresholds reported in his percutaneous system for the median and radial nerve 8-channel FINEs, approximately 120–125 nC [8]. Reduced cuff reshaping and increased encapsulation at the replaced cuff implant sites could cause increased sensory detection thresholds, correlating with a change in selectivity and larger percept areas.

The difference in anode configurations also could affect axon recruitment and percept areas. The 16-channel percutaneous system used monopolar stimulation with an external anode placed near the elbow, whereas the 64-channel iSens system used bipolar stimulation with a large (>2 mm^2^) internal anode. The larger electrode contacts were used as the anode to limit current spread outside the cuff, thus minimizing artifact in recorded EMG. The larger anode also lowered charge density by distributing current over a wider area, preventing anodic stimulation. Previous modeling studies predicted that multipolar stimulation using adjacent or transverse cathode and anode contacts improves selectivity [39, 40], but the effect of anode surface area on selectivity has not previously been evaluated, representing an area for future research. Contrary to expectations, the iSens system’s 16-channel C-FINEs produced larger percepts, suggesting reduced selectivity for this participant. Although the single-sided placement of the large anode strips in the 16-channel C-FINE could have affected current flow, causing more superficial activation for adjacent cathodes compared to transverse cathodes, there was no evidence of this pattern based on percept locations reported by this participant. Further modeling studies are needed to clarify how anode configurations influence axonal recruitment.

Increasing cuff electrode contact density and the number of cuffs increased the number of distinct percept locations across all dissimilarity cutoffs, a pattern also observed when increasing median nerve cuff contacts from 8 to 32 channels. The chosen location dissimilarity cutoff influenced the extent to which that the number of distinct percepts elicited from the 64-channel system exceeded that of the 16-channel system, with more conservative cutoffs yielding more similar cluster counts. Ideally, location dissimilarity cutoff would be chosen based on the overlap between skin mechanoreceptor receptive fields [37]. However, mechanoreceptor receptive fields are highly overlapped in glabrous skin, suggesting that stimulation of distinct, adjacent axons may still evoke seemingly identical percept locations [37]. We recommend selecting a more conservative dissimilarity cutoff, corresponding to lower overlap, to distinguish functionally different hand locations. Investigating the just-noticeable-difference of location position and size could clarify the percept discernibility and better inform location dissimilarity cutoff for cluster analyses.

A limitation of the location dissimilarity metric is that smaller percepts are more likely to be classified as unique even if they are adjacent and functionally represent the same area of the hand, as seen with adjacent percepts evoked by M5 and M6 on the tip of the thumb in the 16-channel system (figure 3(d)). Percepts with nearby centroids and low overlap indicate activation of adjacent axonal populations, given the nerve’s somatotopic organization [11]. Reporting percepts through location drawings [4, 41–44] provides a more exact estimation of axonal recruitment, receptive fields, and overlap compared to selecting centroid points [45, 46], fixed-size regions [47], or subdivided template functional regions [48], but drawing requires motor ability that not all patients possess.

The 64-channel system produced more overlapping percept locations than the 16-channel system, which was expected due to the closer-spaced C-FINE contacts and greater likelihood of recruiting adjacent or identical axonal populations with overlapping receptive fields [10, 11, 37]. Accordingly, two electrode contacts that produced similar, overlapping percept locations could indicate either activation of distinct axons with overlapping receptive fields, activation of the same axons with identical receptive fields, or a combination of both patterns. Such redundancy benefits prosthetic sensory feedback by providing alternative stimulation options in the case of individual contact breakage, facilitating continued sensory feedback. Additionally, applying stimulation paradigms that incorporate percepts at the same location could influence sensation quality and the perception of edges and motion, as seen with intracortical microstimulation in individuals with spinal cord injury [49].

Radial nerve stimulation produced a low number of hand percepts, likely due to nerve anatomy and cuff placement, which limits which contacts can activate the nerve within reasonable current limits [50]. Despite reasonable cross-sectional area for nerve reshaping and acceptable internal pressures [51], the radial nerve was likely uncentered in the C-FINE and did not elongate toward all contacts, limiting axonal recruitment at one end of the cuff. Many radial nerve percepts were proprioceptive or involved upper arm contractions, consistent with radial nerve innervation of motor efferents proximal to the elbow and the susceptibility of sensory afferents to amputation-related degeneration [52–54]. Although the surgery to explant the participant’s percutaneous system and implant the iSens system did not result in motor or sensory deficits [14], radial nerve atrophy due to the original amputation could have affected primary afferents while preserving the motor efferents, contributing to more contractions and proprioceptive sensations in the upper arm [54]. Thus, high-density electrodes are better prioritized for the median and ulnar nerves, which more reliably evoke hand sensations.

We do not claim that this single-participant study will generalize to all individuals with limb loss but do recommend that higher channel systems be focused on median and ulnar nerve implementations. We hypothesize that individuals with higher-level amputations requiring more proximal electrode placements would experience a higher ratio of muscle contractions to cutaneous percepts when stimulating the radial nerve, resulting in less unique sensations on the hand for both low- and high-channel systems. In contrast, because the median and ulnar nerves innervate skin on the hand and forearm muscles, more proximal placements should have less impact on percept size, overlap, or total location clusters, regardless of electrode channel count. Placing electrodes at the brachial plexus, before nerve branching and at a greater cross-sectional area, would likely further reduce the ability to isolate sensory afferents from motor efferents across all three nerves, yielding fewer hand percepts for both low- and high-channel electrodes and minimizing differences in location coverage and clustering between systems.

The ability to selectively steer the electric field across the nerve cross section with high density electrodes represents a significant advantage for neural interfaces, as seen in deep brain stimulation [55], cochlear implants [56], and peripheral nerve electrodes [40]. Increasing stimulation channel count enables more cathode–anode electrode configuration options to precisely target specific areas of the nerve cross section and enable a higher spatial resolution of percepts, though the effects of field steering with 16-channel C-FINEs on percept location remains unevaluated.

Eliciting different types of percepts is key for bidirectional, sensorized prostheses. Modern prosthesis designs incorporate higher densities of pressure, bend, and position sensors [57–61], and mapping each sensor to a distinct percept better mimics the high density of mechanoreceptors and percept locations of the intact hand [37, 62].

### 4.2. Controller performance in 4 DOFs

Overall, increasing the number of intramuscular EMG inputs to a 4-DOF controller resulted in better performance. The added muscle inputs from FPL and ECRB positively affected posture matching success rate, emphasizing that recording from multiple muscle inputs improves the ability to control a virtual hand. FPL plays an important role in controlling thumb movements [63], so the additional FPL input likely enhanced decoding the 4th DOF for thumb abduction/extension-adduction/flexion and contributed to improved posture matching success rates. Similarly, prior work has shown that increasing surface EMG inputs for a pattern recognition control can improve online classification accuracy, completion time, and path efficiency [23].

All three controllers achieved higher path efficiencies than previous 4-DOF regression-based controllers using 6 and 8 TIM inputs, which reported path efficiencies between 20%–30% [17]. The 14-channel controller’s redundant FDS and EDC inputs, which control individual finger motions [64], provided additional information about movement intent to control the virtual hand. Recording from multiple sources that relate to finger flexion and extension supports the development of controllers with independent individual finger movement to better mimic sound hand control [65, 66].

Time-to-target did not significantly differ across controllers, but the 8-channel controller trended toward longer trial durations and more unmatched trials that reached the 30 s trial limit, suggesting slower performance and more time spent attempting to correctly position the virtual hand. All three controllers demonstrated similar initial trial durations to those reported for previously tested 4-DOF regression-based controllers with 6 and 8 intramuscular EMG inputs, between 9–13 s [17], suggesting no speed improvements from the additional channels used in this case study. However, continued practice may reduce time-to-target, as shown in prior work [17]. This also implies that the testing order could impact performance in favor of controllers tested later, despite the participant being blinded to the controller condition during each trial. We tested the 14-channel controller prior to the other controllers during the first two sessions, potentially resulting in lower performance than if tested later after more practice. Further testing with more study participants is needed to separate potential learning effects on performance and determine the relationship between implanted channel count and posture matching speed.

Several groups have explored implanted EMG- and nerve electrode-based classifiers for prosthetic control, which decode movement states for sequential hand and arm movements but do not enable independent, simultaneous joint control [18, 21, 45, 67]. Simultaneous, independent, and proportional prosthetic control has been demonstrated in 2 DOFs [68–70], 3 DOFs [16, 19, 69, 71], and 4 DOFs [17, 35] using implanted or surface EMG inputs. Building on this work, we tested a 4-DOF controller to allow independent, simultaneous control of the wrist and fingers. However, control remains far below the 20 DOFs of a sound hand [26], underscoring the need for increasing controller DOFs.

The participant’s iSens system used 32 EMG channels configured into 16 bipolar pairs, with up to 14 pairs tested for 4-DOF simultaneous, independent, and proportional control. In comparison, another non-percutaneous system for EMG recording, the IMES® System, supports simultaneous, independent, proportional control in up to 3 DOFs with 8 electrodes and, while lacking sensory restoration, remains a promising non-percutaneous approach for intuitive prosthetic control [19]. Similar studies using 8 bipolar intramuscular EMG channels implanted percutaneously in residual muscles and regenerative peripheral nerve interfaces (RPNIs) showed improved classifier performance when including RPNI inputs, reinforcing the value of additional EMG channels from diverse muscle targets [21].

The e-OPRA system, a sensorimotor osseointegrated interface, used up to 12 unipolar epimysial and intramuscular EMG channels, akin to 6 bipolar channels, to sequentially classify six prosthetic movements during home use [18]. While osseointegration improves prosthesis comfort and decreases external bulk [72], the number of conductors that can be fed through the osseointegrated implant currently limits channel count [7, 18]. A hybrid approach combining osseointegration with a wirelessly connected iSens system could enhance comfort while expanding recording and stimulation compatibilities.

Percutaneous systems recording movement-related signals from 2–3 100-channel Utah Slant Electrode Arrays (USEAs) on median and ulnar nerves and 8 intramuscular EMG leads, totaling 32 unipolar EMG channels, have a higher number of recording channels compared to the iSens system [15]. The intramuscular EMG channels showed stable recordings through 502 d post-implant, but the USEAs lost responsive electrodes within weeks to months, limiting chronic use for long-term prosthetic control [15]. Effective high-channel recording systems must ensure long-term stability, as demonstrated in multiple studies using implanted epimysial and intramuscular EMG control [9, 15–18, 21].

Wireless implants enable more channels without the limitations of available skin surface area for percutaneous exit sites [13, 19], supporting the development of more naturalistic prosthetic control with high accuracy, many DOFs, and simultaneous, independent, and proportional control. The development of naturalistic control has implications for improving function for upper limb prosthesis users, who often use compensatory motions [73] and experience reduced function during activities of daily living [74]. Multiple DOF prosthetic control can decrease compensatory motions for upper limb prosthesis users [75] and enable improvements in activities of daily living performance [18]. Although the study presented here did not focus on functional performance, virtual environment controller performance correlates with prosthesis functional performance [76], suggesting that the 14-channel 4-DOF controller would be a strong candidate for future functional testing. As this study only investigated controller performance in a single individual, further virtual and functional testing will be integral for understanding general controller performance trends and optimizing the balance between channel count, myoelectric controller performance, device complexity, and surgical risk.

## 5. Conclusion

This case study demonstrates the benefits of increasing implanted channel count to elicit diverse sensory percept locations across the hand and improve myoelectric controller performance. Eliciting distinct percept locations with a high-density, sensorized prosthesis and developing reliable, high-DOF prosthetic control moves bidirectional prostheses toward resemblance to the sound hand. The continued investment and effort toward increasing implanted channel count can provide value for individuals with upper limb loss as well as for other neuromuscular conditions.

## Data Availability

The data cannot be made publicly available upon publication because they are not available in a format that is sufficiently accessible or reusable by other researchers. The data that support the findings of this study are available upon reasonable request from the authors.
